# Endoscopic Findings in Patients Presenting Dyspepsia: A Population-Based Study in Mashhad, North East of Iran

**DOI:** 10.34172/mejdd.2025.402

**Published:** 2025-01-31

**Authors:** Mina AkbariRad, Abdollah Firoozi, AmirAli Moodi Ghalibaf, Hassan Mehrad-Majd, Bahram Kangi, Ali Beheshti Namdar

**Affiliations:** ^1^Department of Internal Medicine, Faculty of Medicine, Mashhad University of Medical Sciences, Mashhad, Iran; ^2^Student Research Committee, Birjand University of Medical Sciences, Birjand, Iran; ^3^Clinical Research Development Centre, Ghaem Hospital, Faculty of Medicine, Mashhad University of Medical Sciences, Mashhad, Iran

**Keywords:** Upper gastrointestinal endoscopy, Dyspepsia, Peptic ulcer, Gastrointestinal malignancies

## Abstract

**Background:**

The present study aimed to evaluate the endoscopic findings of patients with dyspepsia in Ghaem Hospital, Mashhad, Iran.

**Methods:**

This cross-sectional study collected endoscopic findings in patients with dyspepsia, including epigastric pain or heartburn, postprandial fullness, and early satiety from Ghaem Hospital from 2019 to 2020.

**Results:**

Totally, 743 patients were studied, and 42.3% (n=314) were male. The mean age was 46 years. Among participants, 85.6% (n=636) were included in the functional dyspepsia group, and the rest were included in the organic dyspepsia group. In the organic dyspepsia group, the highest frequency was related to peptic ulcer disease, with 7.2% (n=53). Moreover, the most common complaint was epigastric pain and heartburn. No significant association was found between comorbidities (*P*=0.083), smoking, and sex (*P*=0.532) with the risk of organic dyspepsia.

**Conclusion:**

Dyspepsia is not necessarily accompanied by other comorbidities. The most mentioned chief complaint was epigastric pain or heartburn. Functional dyspepsia was the most common diagnosis in patients with dyspepsia, and the peptic ulcer was the leading cause of organic dyspepsia. There was no difference in the underlying cause of dyspepsia in the sexes.

## Introduction

 Dyspepsia is a prevalent gastrointestinal (GI) complaint that reduces people’s quality of life and productivity. Also, it imposes enormous costs on healthcare systems.^1-3^ There is a varied prevalence reported for dyspepsia from 1.8% to 57%, considering different criteria, especially in women, smokers, and people taking non-steroidal anti-inflammatory drugs (NSAIDs).^4-6^ Specifically, it has been reported that the prevalence of dyspepsia is relatively high in Iran.^7^ Different risk factors have been reported for causing dyspepsia, such as anxiety, depression, NSAID usage, smoking, and *Helicobacter pylori *infection.^8-12^ These risk factors generally vary in Eastern and Western societies due to the differences in natural microbial flora and dietary habits.^5^ Two main classes of dyspepsia are organic and functional dyspepsia.Approximately 70% to 80% of patients do not show any organic pathology in their endoscopic results supporting their symptoms and will be categorized as functional dyspepsia.^13,14^ According to ROME IV criteria, functional dyspepsia is divided into epigastric pain syndrome, which is for symptoms like epigastric pain or heartburn, postprandial distress syndrome which consists of postprandial fullness or early satiety, or overlapping of both.^5,13^

 Functional and organic dyspepsia can be differentiated by endoscopy of the upper gastrointestinal (GI) tract, which allows the physician to remove GI mucosa or prepare a biopsy from the suspicious mass for a pathological examination.^1,5,10,15-17^ Alternatively, in patients with dyspepsia, less than 10% of patients have GI ulcers, and less than 1% have GI malignancies,^14,18^ however, according to the high prevalence of precancerous lesions in patients with chronic disease, upper endoscopy, and gastric mapping sampling for the detection of these lesions is recommended in intermediate-risk to high-risk areas.^19,20^ Therefore, performing upper endoscopy in all patients with dyspepsia is neither reasonable nor possible.^21,22^ A study conducted in 2014 found that the cost of finding any upper GI malignancy in patients with dyspepsia is more than $80 000 (per diagnosis), so a selective approach is preferred for dealing with these patients even in developed countries.^23,24^ Thus, doctors consider alarm symptoms in their patients to thoroughly examine the upper GI tract by endoscopy, given that there is a possibility of underlying GI cancers.^24^ Alarm symptoms include age over 55, melena or hematemesis, dysphagia or odynophagia, persistent nausea, unexplained weight loss, family history of GI cancers, palpable mass on abdominal examination, and evidence of an unexplained iron deficiency anemia.^15,24-26^

 Despite the administration of various dyspepsia cases, there is still a lack of studies on the prevalence, predisposing factors, frequency of symptoms, and complications of dyspepsia in the eastern population of Iran until the time of this study. Therefore, this study investigates the aforementioned characteristics of dyspepsia in Ghaem Hospital of Mashhad, Iran. The results can be used for further studies to improve dietary habits and healthcare policies, such as promoting increased consumption of fruits and vegetables through community-based educational programs. Additionally, the findings could guide healthcare policies by advocating for improved access to nutritious foods in underserved areas and implementing regulations that limit the marketing of unhealthy food options to vulnerable populations. These strategic improvements could ultimately lead to better public health outcomes and a reduction in diet-related diseases

## Materials and Methods

###  Setting, Sampling, and Selection Criteria

 This cross-sectional study was conducted using a census sampling method at Ghaem Hospital, Mashhad University of Medical Sciences, Mashhad, Iran, on patients with dyspepsia who underwent upper endoscopy in 2019-2020. All patients aged 18 to 80 years who presented with epigastric pain or heartburn, postprandial fullness, and early satiety were eligible to be included in the study. Patients with pain in areas other than the epigastrium, patients undergoing endoscopy with complaints other than dyspepsia, such as GI bleeding or gastroesophageal reflux, and patients aged under 18 or over 80 years were excluded.

###  Study Procedures

 The endoscopic data of patients who had been symptomatic for at least 6 months prior to administration and had symptoms of dyspepsia for the past 3 months were recorded. Confounding exposures such as age, sex, underlying diseases, smoking, and medications used by patients were asked in interviews or extracted from patients’ previous records, and the frequency of each of them was evaluated. Further comparisons were conducted between the two sexes. The outcomes were divided into two general categories: Functional dyspepsia, in which no specific underlying factor was found to justify patients’ symptoms in endoscopy, and organic dyspepsia, which explains the specific underlying cause of the patient’s symptoms.^27^ Organic dyspepsia was divided into underlying reasons that justified the patients’ symptoms, and the frequency and percentage of each were calculated and recorded. Specific cases, such as GI malignancies, were recorded and reported case-by-case. Additionally, the frequency of chief complaints was evaluated and was compared in functional and organic dyspepsia groups.

###  Statistical Analysis

 Demographic characteristics and clinical conditions, disease causes, and endoscopic findings of patients were recorded. The frequency of each was measured separately. The collected data were finally entered into IBM SPSS software version 22 and statistically analyzed. Nominal variables were presented as percentages and counts and were analyzed using chi-square tests or Fisher’s test. Ordinal variables were expressed by mean ± standard deviation and were analyzed using t-test or Kruskal–Wallis test. *P* < 0.05 was considered statistically significant. Furthermore, a ROC curve with a sensitivity of 48% and a specificity of 63% was carried out to calculate the age limit for endoscopic studies in dyspepsia.

## Results

###  Demographic Characteristics

 Following inclusion and exclusion criteria, 743 patients participated in the study. Of these, 42.3% were male, and the mean age was 46.08 ± 14.97 years. Of the patients, 144 (19.4%) were smokers or addicts (64.8% were smokers, and 35.2% were opium addicts based on their self-report). 83% of patients had no other diseases. Hypertension, hyperlipidemia, hypothyroidism, and diabetes mellitus (DM) were the most common comorbidities (Table 1). Also, dyspepsia-related comorbidities were insignificant between functional and organic dyspepsia (P = 0.083). Considering drug history, 83% of patients did not take any medications. The most common medications were atorvastatin, losartan, aspirin, levothyroxine, and metformin (Table 1).

**Table 1:** Total frequency of comorbidities and specific frequencies in functional and organic dyspepsia, along with medications prescribed for each category

**Comorbidities**	**Total frequency (%)**	**Frequency in functional dyspepsia (%)**	**Frequency in organic dyspepsia (%)**
Without comorbidity	617 (83)	527 (82.9)	90 (84.1)
Hypertension	10 (1.3)	10 (1.6)	0
Hyperlipidemia	31 (4.2)	25 (3.9)	6 (5.6)
Hypertension and hyperlipidemia	32 (4.3)	27 (4.2)	5 (4.7)
Diabetes mellitus, hypertension, and hyperlipidemia	26 (3.5)	20 (3.1)	6 (5.6)
Hypothyroidism	27 (3.6)	27 (4.2)	0
**Medications**		**Frequency (%)**	
No medication		617 (83)	
Anti-hypertensives (e.g., losartan)		10 (1.3)	
Atorvastatin		31 (4.2)	
Anti-hypertensives and atorvastatin		32 (4.3)	
Metformin, aspirin, anti-hypertensives, and atorvastatin		26 (3.5)	
Levothyroxine		27 (3.6)	

 The most common chief complaint that patients presented with was epigastric pain or heartburn, with a frequency of 41.3%. Postprandial fullness was the second most common complaint, with a frequency of 35.7%. Additionally, early satiety was a minor chief complaint (Table 2). The frequency of chief complaints in the two groups of functional dyspepsia and organic dyspepsia was calculated separately, and no significant difference was observed between them based on the Chi-square test (*P* = 0.086).

**Table 2:** Chief complaints frequency in all the patients and separately in functional and organic dyspepsia

**Chief complaints**	**Total frequency (%)**	**Frequency in functional dyspepsia**	**Frequency in organic dyspepsia**
Epigastric pain or heartburn	41.3	42.8	32.7
Postprandial Fullness + bloating	35.7	34.3	43.9
Epigastric pain or heartburn + Postprandial fullness	16.4	16.2	17.8
Epigastric pain or heartburn + postprandial fullness + bloating	5.2	5.7	2.8
Epigastric pain or heartburn + early satiety	1.3	1.1	2.8

###  Endoscopic Findings

 The upper GI tract endoscopic results were divided into two major categories: functional dyspepsia and organic dyspepsia. Cases of functional dyspepsia had no findings to justify their symptoms on endoscopy. The number of patients with functional dyspepsia was 636 (85.6%), and the number of patients with organic dyspepsia was 107 (14.4%). Underlying factors seen on endoscopy included gastric, duodenal, or esophageal ulcers, duodenal erosions, hyperplastic polyps, and esophagitis. The frequency of each underlying problem is shown in Table 3 in detail.

**Table 3:** Frequency of underlying reasons for dyspepsia in endoscopic results and their comparisons in two sexes

**Underlying factor**		**Frequency (%)**	**Frequency in men (%)**	**Frequency in women (%)**
Functional dyspepsia		85.6	87.6	84.1
Organic dyspepsia	Duodenal ulcer	4.2	3.2	4.9
	Gastric ulcer	3.0	2.5	3.3
	Gastric hyperplastic polyps	2.7	1.9	3.3
	Duodenal erosion	1.3	1.3	1.4
	Esophageal ulcer	0.8	1.3	0.5
	Esophagitis	0.8	1.0	0.7
	Gastric adenocarcinoma	0.5	0.6	0.5
	Incomplete intestinal metaplasia	0.5	0.0	0.9
	Gastric ulcer - duodenal ulcer	0.4	0.3	0.5
	Esophageal squamous cell carcinoma	0.1	0.3	0.0

**Figure 1:**
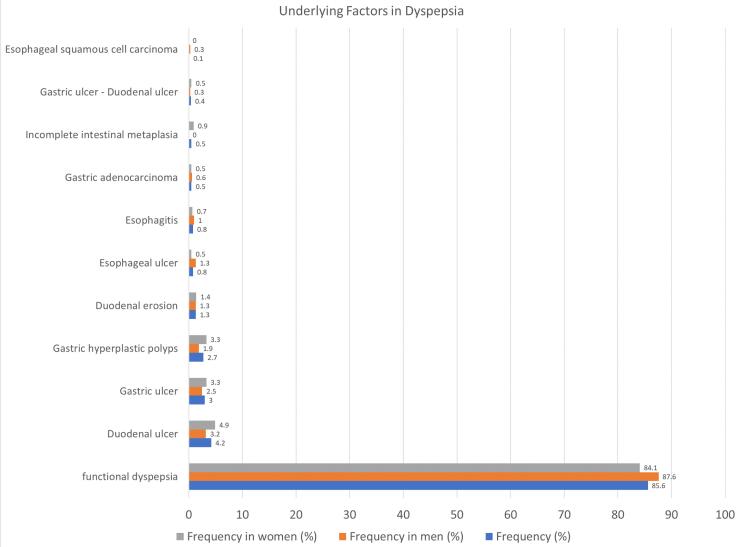
Frequency of underlying reasons for dyspepsia in endoscopic results and their comparisons in two sexes

 In endoscopy, 20 patients (2.7%) had polyps. All of these cases were hyperplastic polyps in the pathology report, and there was no finding in favor of malignancy. Moreover, a biopsy mass was prepared if a mucosal change or suspicious mass was seen during the endoscopy. Among these patients, four cases (0.5%) of incomplete intestinal metaplasia, four cases (0.5%) of gastric adenocarcinoma, and one (0.1%) with esophageal squamous cell carcinoma were reported in the pathology report (Figure 1).

 With regard to the Fisher test, there was no statistically significant difference between men and women in their endoscopic results (*P* = 0.532).

**Figure 2:**
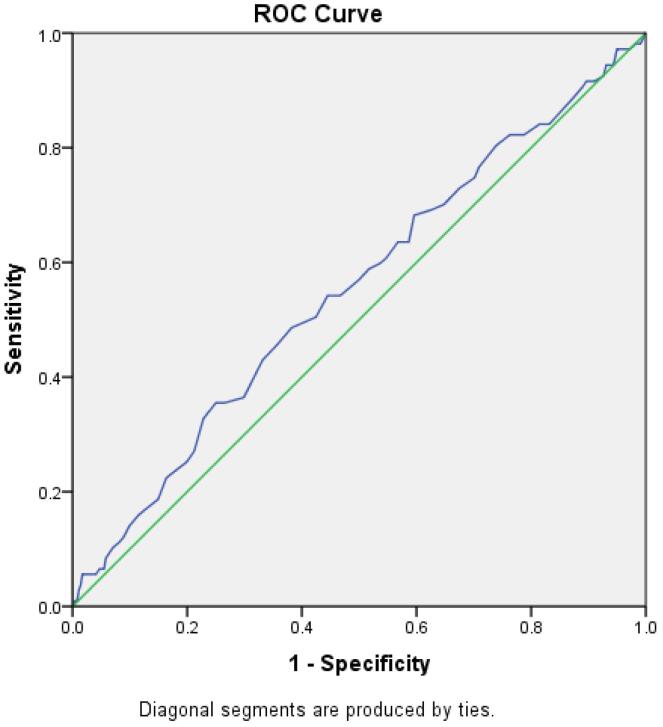
ROC curve for age threshold calculation

 The age threshold as an alarm sign for organic dyspepsia was concluded with a ROC curve with a sensitivity of 48% and a specificity of 63%. The age was calculated to be 50 years (Figure 2).

## Discussion

 In Iran, endoscopic findings in patients with dyspepsia have not been studied in detail. This study was conducted to investigate the underlying causes of dyspepsia in Iranian society due to the high prevalence of GI diseases such as dyspepsia and their consequences on mental health and the economic burden of the country’s health care system. In detail, it could lead to anxiety, depression, low quality of life, and somatization.^28^ Based on our findings, functional dyspepsia was the most frequent category among patients. Considering organic dyspepsia, peptic ulcers were the main underlying factor. Additionally, comorbidities, sexes, and medications did not significantly affect dyspepsia.

 Regarding our results, the most common complaint of the patients was epigastric pain syndrome like epigastric pain or heartburn. It is consistent with a study conducted in Qatar in 2020 on 733 people, where the most common complaint was epigastric pain.^29^ However, in some other investigations in other countries, postprandial distress syndrome such as postprandial fullness and early satiety, occurred two times more than EPS.^30^ It is important to note that dietary habits could have a significant role in this issue, as some diets can potentially increase the risk of dyspepsia, and some of them could be protective.

 Among the underlying causes of dyspepsia, the most common was functional dyspepsia. Other studies have reported the same result as well. In a survey conducted in 2014 on 282 patients in Brazil by Faintuch and colleagues, 66% of patients had functional dyspepsia.^1^ Another retrospective study in Kenya found that the highest frequency of endoscopic findings was related to functional dyspepsia with perfectly normal mucosa.^31^ Additionally, there was no significant difference between the results of men and women. Nevertheless, several studies have mentioned that dyspepsia is more frequent in women than men.^32,33^ The relationship between dyspepsia and sex is not fully understood, and further investigations are needed.^32^ Comorbidities and systemic diseases such as hypertension or diabetes could not be significantly associated with functional or organic dyspepsia. By the way, psychological disorders like depression, anxiety, and stress can accompany dyspepsia very often.^34,35^

 In summary, our findings contribute to the understanding of dyspepsia by re-affirming functional dyspepsia as a predominant cause while highlighting the complexity of its relationship with sex and comorbid conditions. The significant role of psychological factors in dyspeptic presentations calls for an integrated approach to treatment that addresses both physical and mental health aspects. Future research should continue to explore these relationships to enhance our understanding of dyspepsia’s multifaceted nature and improve patient outcomes through tailored interventions.

 In our investigations, peptic ulcer (duodenal and gastric) was the primary cause of organic dyspepsia. Faintuch and colleagues’ study demonstrated the same result with higher frequencies of duodenal and gastric ulcers.^1^ Also, in older or recent studies conducted in different societies, the most common pathological result in endoscopy of patients with organic dyspepsia was peptic ulcers.^31,36,37^ However, various researchers have reported other endoscopic findings, such as esophagitis, gastritis, and duodenitis, as the most common underlying cause of organic dyspepsia.^29,31,38^ The high percentage of organic dyspepsia in this study may be due to the consideration of cases of GI mucositis, such as gastritis, as organic dyspepsia.^29^ These cases were considered functional dyspepsia in our experiment because they did not explain patients’ symptoms. Consequently, few cases of GI malignancies were found in our endoscopic results. GI cancers were also an uncommon finding among patients of other research.^1,31,38^ This discrepancy may stem from differences in patient demographics, geographical factors, or diagnostic criteria employed in different studies. The high incidence of organic dyspepsia observed in our study could also be influenced by our classification criteria, particularly regarding GI mucositis. Additionally, it suggests that while malignancies should always be considered in the differential diagnosis of dyspepsia, they are not typically the primary cause in most cases.

 One of the alarm signs for endoscopic investigations in dyspepsia is age burden, which in our study was evaluated to be more than 50 years. In another study, the onset age of GI malignancies was 55 years, and the risk age for organic dyspepsia was 48 years,^1^ which is very close to our results. Moreover, the frequency of malignancies was 1.8%, approximately three times higher than our study.^1^ The higher percentage of GI malignancies may be due to the smaller sample size of this study compared with ours. Malignancies are negligible without red flags and if the age is under 50 years, there is no need to do an upper GI endoscopy.^39^ Future research should continue to refine these age-related thresholds and explore additional clinical indicators that may assist in distinguishing between functional and organic causes of dyspepsia. This could ultimately lead to more targeted and effective management strategies tailored to individual patient profiles.

 One of the strengths of our study is the high statistical population, which is higher compared with similar studies. On the other hand, Ghaem Hospital, Mashhad University of Medical Sciences, Mashhad, Iran, is a referral center where patients from all over the east and some central provinces of Iran come over. This will increase the dispersion of patients in the study, and the data will be more reliable. Moreover, the endoscopy center of Ghaem Hospital is equipped with video endoscopy techniques, which significantly increases physicians’ diagnostic power. Considering the limitations, it is possible that patients may give incorrect information during our interviews. Also, they may refuse to provide adequate information for personal reasons in some cases.

 It is possible that anxiety issues could lead to functional dyspepsia due to high environmental stressors.^40^ In future studies, researchers can consider the associations between psychiatric disorders such as anxiety, depression, and stress with the possibility of functional dyspepsia and investigate the reason for the higher prevalence of functional dyspepsia in Iranian society, strongly suggested to include the population from different regions of the country which could represent better insight of the problem and cover demographic conditions. Also, we suggest that a cause-and-effect relationship between confounding factors and dyspepsia can be discovered by changing the study method to studies such as cohort. Furthermore, future studies can use a larger sample size and investigate on a larger scale to make the results more generalizable to the entire Iranian community.

## Conclusion

 According to our findings, dyspepsia is not necessarily accompanied by other comorbidities, and most patients have no other diseases. Also, the chief complaint that was mentioned most was epigastric pain or heartburn. The symptoms did not show a significant difference between functional and organic dyspepsia.

 Functional dyspepsia was the most common diagnosis in patients with dyspepsia, while the frequency of GI-related malignancies was low. Moreover, the peptic ulcer was the leading cause of organic dyspepsia. There was no difference in the underlying cause of dyspepsia between men and women.

 Due to the low prevalence of malignancies, it is better not to conduct upper GI endoscopy in all patients with symptoms of dyspepsia. Along with the alarm symptoms, it is recommended that patients with dyspepsia older than 50 years old undergo upper GI endoscopy.
